# Supplementary material for: Networked partisanship and framing: A socio-semantic network analysis of the Italian debate on migration
Source: PLoS One. 2021 Aug 26;16(8):e0256705. doi: 10.1371/journal.pone.0256705 (PMC8389375; doi:10.1371/journal.pone.0256705)
Supplement: S1 Appendix — (PDF) [file pone.0256705.s001.pdf]

Networked partisanship and framing: a socio-semantic network analysis of the Italian debate on migration - S1 Appendix

Tommaso Radicioni*[1,2], Fabio Saracco[2], Elena Pavan[3], Tiziano Squartini[2]

1 Scuola Normale Superiore, P.zza dei Cavalieri 7, 56126 Pisa (Italy)
2 IMT School for Advanced Studies, P.zza S. Francesco 19, 55100 Lucca (Italy)
3 University of Trento, via Verdi 26, 38122 Trento (Italy)

*tommaso.radicioni@sns.it

S1 Appendix. Overview of events related to the Italian debate on migration. Since the beginning of 2014, when the numbers of migrants attempting to enter the European Union massively increased, the refugees crisis is a hot-topic widely discussed on the European mainstream media [1]. The prominence of this topic has been increased also due to the rise of European political parties fuelling xenophobic or anti-migration policies or narratives that have built a rhetorical construction of immigrants and refugees as a dangerous threat for national security.

In June 2018, the two (initially competing) parties emerged as winners after the Election day, i.e. the Five Stars Movement and the League, formed a new government. In the self-proclaimed ‘government of change’, the League party, albeit the minority partner in the coalition, successfully succeeded in influencing the political agenda of the government on several topics at the center of its political project, e.g. the migration policies. One of most debated decisions taken by Matteo Salvini was the closure of the Italian ports to the NGOs boats rescuing migrants in the Mediterranean Sea. This political initiative not only took the tangible forms of entry bans into Italian ports or seizing of several rescue vessels but also in the approval of two decrees on security - the second one called Second Security Act (‘Decreto Sicurezza Bis’) was approved in June 2019 - the increased the difficulties, for asylum seekers, to request the residence permits issued for humanitarian reasons and introduced fines for NGOs active in migrants rescuing activities. On 19 August, approximately 14 months after its formation, the governement fell upon the initiative of Matteo Salvini that submitted a no-confidence motion against the Prime Minister Giuseppe Conte. Few weeks later, the M5S formed a new government with the Democratic Party (and other minor forces of the Italian left-wing parties) while the League went back to the opposition. The new government immediately presented a different attitude towards the NGOs involved in search-and-rescue operations: on 26 October, the new Minister of the Internal Affairs, Luciana Lamorgese, meet the organizations to discuss their activities.

The other major political events of interest for our analysis are 1) the European election on 26 May 2019 and 2) the entering of the rescue vessel Sea-Watch 3 into the Italian territorial waters, without permission, at end of June 2019. The former one is a significant electoral event since the League became the most voted Italian party, with 34.1% of the vote share (that reversed the political result of the 2018 general elections, after which the Five Stars Movement emerged with a similar percentage of preferences); the latter one is one of the most debated events about migration before the fall of the government: after two weeks sailing, on 29 June 2019, the rescue vessel Sea-Watch 3 entered the Italian territorial waters and its captain Carola Rackete was arrested. Matteo Salvini, the back-then Minister of the Interior, accused Rackete of hitting an

Italian patrol boat which tried to intercept the Sea-Watch 3 before docking. At the same time, Rackete was under investigation by Italian authorities for alleged criminal activities concerning undocumented activities in search-and-rescue operations. After a brief detention under house arrest, she was released by an Italian court ruling that she acted to protect the safety of the passengers.

S1 Table provides a brief overview of the most relevant political and mediatic events concerning the Italian Twittersphere discussion about migration. (see also [2] for an overview of the Italian discussion on migration issues across the period August 2018-July 2019).

References

1. Berry M, Garcia-Blanco I, Moore K. Press coverage of the refugee and migrant crisis in the EU: A content analysis of five European countries. United Nations High Commissioner for Refugees; 2016.

2. Vilella S, Lai M, Paolotti D, Ruffo G. Immigration as a Divisive Topic: Clusters and Content Diffusion in the Italian Twitter Debate. Future Internet. 2020;12(10). doi:10.3390/fi12100173.
