# Supplementary material for: Networked partisanship and framing: A socio-semantic network analysis of the Italian debate on migration
Source: PLoS One. 2021 Aug 26;16(8):e0256705. doi: 10.1371/journal.pone.0256705 (PMC8389375; doi:10.1371/journal.pone.0256705)
Supplement: S2 Appendix — (PDF) [file pone.0256705.s002.pdf]

Networked partisanship and framing: a socio-semantic network analysis of the Italian debate on migration - S2 Appendix

Tommaso Radicioni*[1,2], Fabio Saracco[2], Elena Pavan[3], Tiziano Squartini[2]

1 Scuola Normale Superiore, P.zza dei Cavalieri 7, 56126 Pisa (Italy)
2 IMT School for Advanced Studies, P.zza S. Francesco 19, 55100 Lucca (Italy)
3 University of Trento, via Verdi 26, 38122 Trento (Italy)

*tommaso.radicioni@sns.it

S2 Appendix. Description of the main hashtags. In our analysis, hashtags might be obscure to a reader who is not familiar with the Italian language and with societal and political references related with them. For this reason, in S2 Table we provide a translation of the main Italian hashtags along with a brief description of the Italian political and societal context.
