# Supplementary material for: Networked partisanship and framing: A socio-semantic network analysis of the Italian debate on migration
Source: PLoS One. 2021 Aug 26;16(8):e0256705. doi: 10.1371/journal.pone.0256705 (PMC8389375; doi:10.1371/journal.pone.0256705)
Supplement: S3 Appendix — (PDF) [file pone.0256705.s003.pdf]

Networked partisanship and framing: a socio-semantic network analysis of the Italian debate on migration - S3 Appendix

Tommaso Radicioni*[1,2], Fabio Saracco[2], Elena Pavan[3], Tiziano Squartini[2]

1 Scuola Normale Superiore, P.zza dei Cavalieri 7, 56126 Pisa (Italy)
2 IMT School for Advanced Studies, P.zza S. Francesco 19, 55100 Lucca (Italy)
3 University of Trento, via Verdi 26, 38122 Trento (Italy)

*tommaso.radicioni@sns.it

S3 Appendix. Bipartite networks projection and validation. This section provides a brief overview of the algorithm we have implemented to project our bipartite networks on a single layer (be it the one of verified users or the one of the hashtags). Generally speaking, this procedure outputs a monopartite projection by linking any two nodes, belonging to the same layer, if the number of their common neighbors is statistically significant; it can be summarized into three steps.

First, a measure quantifying the degree of similarity between two nodes is needed. Given any two nodes α and β of the same layer $\perp$, their similarity is provided by the total number of co-occurrences, i.e. the number of common neighbors $V^*_{\alpha\beta}$, computable as $V^*_{\alpha\beta} = \sum_{j=1}^{N_\top} V^j_{\alpha\beta} = \sum_{j=1}^{N_\top} m_{\alpha j} m_{\beta j}$. The term $V^j_{\alpha\beta}{=}m_{\alpha j} m_{\beta j}$ denotes the 'single' common neighbor, defined by the nodes α and β with j belonging to the opposite layer; its value is 1 if nodes α and β share node j as a common neighbor and 0 otherwise.

Second, the statistical significance of any two nodes similarity needs to be quantified. To this aim, observations have to be compared against a proper null model that can be defined within the mathematical framework of the so-called Exponential Random Graphs. This framework is based on a very general principle rooted in statistical physics [1], prescribing to employ the conservative benchmarks that are induced by the maximization of Shannon entropy. In mathematical notation, given the ensemble $\mathcal{M}$ of networks and the probability $P(\mathbf{M})$ of occurrence of a network $\mathbf{M} \in \mathcal{M}$, the Shannon entropy is

$$S = -\sum_{\mathbf{M}\in\mathcal{M}} P(\mathbf{M}) \ln P(\mathbf{M}) \tag{1}$$

where the sum runs over the set of all possible bipartite graphs with, respectively, $N_\top$ nodes on the $\top$ layer and $N_\perp$ nodes on the $\perp$ layer - as the real network system $\mathbf{M}^*$. As the entropy-maximization procedure is carried out in a constrained framework, let us define the constraints of the Bipartite Configuration Model (BiCM) [2], i.e. the null model adopted in the present paper. In this specific model, the ensemble average of the degrees of users and hashtags (i.e. $k^*_i = \sum_\alpha m_{i\alpha}$, $\forall\, i$ and $h^*_\alpha = \sum_i m_{i\alpha}$, $\forall\, \alpha$, respectively) are considered as fixed. Upon introducing the Lagrange multipliers $\boldsymbol{\theta}$ and $\boldsymbol{\eta}$ to enforce the proper constraints and ψ to ensure the normalization of the probability, the recipe prescribes to maximize the Lagrangian function

$$\mathcal{L} = S - \psi\left[1 - \sum_{\mathbf{M}\in\mathcal{M}} P(\mathbf{M})\right] - \sum_{i=1}^{N_\top} \theta_i [k^*_i - \langle k_i\rangle] - \sum_{\alpha=1}^{N_\perp} \eta_\alpha [h^*_\alpha - \langle h_\alpha\rangle] \tag{2}$$

with respect to $P(\mathbf{M})$. This leads to:

$$P(\mathbf{M}|\boldsymbol{\theta},\boldsymbol{\eta}) = \prod_{i=1}^{N_\top}\prod_{\alpha=1}^{N_\perp} p_{i\alpha}^{m_{i\alpha}}(1-p_{i\alpha})^{1-m_{i\alpha}} \tag{3}$$

where $x_i \equiv e^{-\theta_i}$, $y_\alpha \equiv e^{-\eta_\alpha}$ and the quantity $p_{i\alpha} \equiv \frac{x_i y_\alpha}{1+x_i y_\alpha}$ is the probability that user i and hashtag α are connected (i.e. that $m_{i\alpha}=1$).

Links independence under the BiCM implies that 1) the presence of a co-occurrence (i.e. $m_{\alpha j}m_{\beta i}=1$) can be described as the outcome of a Bernoulli trial whose probability reads $f_{\text{Ber}}(m_{\alpha j}m_{\beta i}=1)=p_{\alpha j}p_{\beta j}$ and that 2) the term $V_{\alpha\beta}$ is the sum of independent Bernoulli trials, each one characterized by a different probability. The behavior of such a random variable is described by the Poisson-Binomial (PB) distribution. Thus, quantifying the statistical significance of the similarity of nodes α and β amounts at computing

$$\text{p-value}(V^*_{\alpha\beta}) = \sum_{V_{\alpha\beta}\geq V^*_{\alpha\beta}} f_{\text{PB}}(V_{\alpha\beta}); \tag{4}$$

this procedure is repeated for each pair of nodes, hence obtaining $\binom{N_\perp}{2}$ p-values.

Third, in order to understand which p-values are significant, a validation procedure for testing simultaneously multiple hypotheses is needed. The choice of the present paper has been directed towards the False Discovery Rate (FDR) [3] which prescribes to, first of all, sort the $\binom{N_\perp}{2}$ p-values in increasing order, i.e. $\text{p-value}_1 \leq \ldots \leq \text{p-value}_n$, and, then, identify the largest integer $\hat{i}$ satisfying the condition $\text{p-value}_{\hat{i}} \leq \frac{\hat{i}t}{n}$, where t represents the single-test significance level (in our case, it is set to 0.01). All p-values that are less than, or equal to, $\text{p-value}_{\hat{i}}$ are, thus, kept, meaning that all the corresponding node pairs are linked in the resulting monopartite projection.

References

1. Cimini G, Squartini T, Saracco F, Garlaschelli D, Gabrielli A, Caldarelli G. The statistical physics of real-world networks. Nature Reviews Physics. 2019;1(1):58–71. doi:10.1038/s42254-018-0002-6.

2. Saracco F, Clemente RD, Gabrielli A, Squartini T. Randomizing bipartite networks: the case of the World Trade Web. Scientific Reports. 2015;5:10595. doi:10.1038/srep10595.

3. Benjamini Y, Hochberg Y. Controlling the false discovery rate: a practical and powerful approach to multiple testing. J R Stat Soc B. 1995;57(1):289–300.
