# Supplementary material for: Networked partisanship and framing: A socio-semantic network analysis of the Italian debate on migration
Source: PLoS One. 2021 Aug 26;16(8):e0256705. doi: 10.1371/journal.pone.0256705 (PMC8389375; doi:10.1371/journal.pone.0256705)
Supplement: S4 Appendix — (PDF) [file pone.0256705.s004.pdf]

Networked partisanship and framing: a socio-semantic network analysis of the Italian debate on migration - S4 Appendix

Tommaso Radicioni*[1,2], Fabio Saracco[2], Elena Pavan[3], Tiziano Squartini[2]

1 Scuola Normale Superiore, P.zza dei Cavalieri 7, 56126 Pisa (Italy)
2 IMT School for Advanced Studies, P.zza S. Francesco 19, 55100 Lucca (Italy)
3 University of Trento, via Verdi 26, 38122 Trento (Italy)

*tommaso.radicioni@sns.it

S4 Appendix. Analysis of mesoscale network structures. The Louvain algorithm [1] has been run on the monopartite projections of the networks of users to detect the presence of communities. This algorithm works by searching for the partition attaining the maximum value of the modularity function Q, i.e.

$$Q = \frac{1}{2L}\sum_{i,j}\left[a_{ij} - \frac{k_i k_j}{2L}\right]\delta_{c_i,c_j} \qquad (1)$$

a score function measuring the optimality of a given partition, by comparing the empirical pattern of interconnections with the one predicted by a properly-defined benchmark model. In the formula above, a_{ij} is the generic entry of the network adjacency matrix $\mathbf{A}$, the factor $\frac{k_i k_j}{2L}$ is the probability that nodes i and j establish a connection according to the Chung-Lu model, c is the N-dimensional vector encoding the information carried by a given partition (the i-th component, c_i, denotes the module to which node i is assigned) and the Kronecker delta δ_{c_i,c_j} ensures that only the nodes within the same modules provide a positive contribution to the sum. For the present analysis, as already pointed out in [2], a reshuffling procedure has been applied to overcome the dependence of the original algorithm on the order of the nodes taken as input. In order to prevent the modularity function from being stuck in a local maximum, the Louvain algorithm is repeated 1.000 times which is a number of runs ensuring the algorithm stability. In order to better explore the solution space of the modularity function Q, a node-reshuffling procedure has been performed: in the first run, the value of the modularity function Q is computed by employing the formula described in Eq. 1 and its value is stored alongside with the initial partition; in the following runs, the order of the nodes to be taken as input by the Louvain algorithm is reshuffled and the value of the modularity function Q is computed again. If the new value of Q is greater than the one obtained in the previous run, both the new value and the new partition are stored; otherwise, the order of nodes is reshuffled again and the procedure is repeated.

Regarding the k-core and the core-periphery decompositions, we briefly describe our approach below. While a proper core-periphery detection can be carried out by adopting the method proposed in [3] and prescribing to search for the network partition minimizing the surprise score function, the presence of a densely connected subgraph can be inspected in a simpler way, i.e. via the k-core decomposition. A k-core is a subgraph whose nodes have a degree whose value is at least k and can be revealed in an iterative fashion (i.e. deleting nodes with degree less than k). A node is said to have 'coreness' c if it belongs to a c-core but not to any $(c+1)$-core.

An interesting phenomenon that is observed when analysing monthly semantic networks is the co-existence of different types of mesoscopic structures. As pointed out in other works [4], communities are found to co-exist with k-shells in several systems: in particular, the innermost k-shells is frequently sub-divided into (a small number) of communities further partitioning it. These sub-communities play an important role: within our partisan communities-induced semantic networks, this role has been interpreted as a natural division of the most debated topics animating the same 'global' discussion. Running the Louvain community detection algorithm on the innermost shell (reported on the right in Fig. 5 and Fig. 6) indeed reveals the existence of these sub-communities. For instance, the core of the DX community displays two distinct sub-communities: one of the clusters concerns a general discussion on the regulation of the migration flows and the behavior of Italian institutions in handling migrants - e.g. hashtags like *#braccianti* ('day labourer'), *#permessi* ('permits') and *#confisca* ('confiscation' - appear); the second one concerns the Sea-Watch 3 crisis and the opposition to its entrance into the Italian ports (e.g. hashtags like *#blocconavale* ('ship block'), *#iostoconsalvini* ('I stand with Salvini') and *#salvininonmollare* ('Salvini don't give up') appear).

Remarkably, the nodes connecting the thematic clusters act as 'bridges' and represents more general hashtags about migration policies (e.g. *#salvini* and *#libia*). In this way, the picture of the list of hashtags with largest betweenness centrality can be further refined upon looking at the innermost k-shells. As an example, let us consider the DX-induced semantic network for the month of July 2019: the keywords playing the role of bridges read *#salvini*, *#capitana* (an untranslatable hashtags referring to Carola Rackete as the captain of the rescue vessel Sea Watch 3) and *#libia*, a signal that these hashtags are included into several discussions within this partisan community.

References

1. Blondel VD, Guillaume JL, Lambiotte R, Lefebvre E. Fast unfolding of communities in large networks. Journal of Statistical Mechanics: Theory and Experiment. 2008;2008(10):P10008. doi:10.1088/1742-5468/2008/10/p10008.

2. Becatti C, Caldarelli G, Lambiotte R, Saracco F. Extracting significant signal of news consumption from social networks: the case of Twitter in Italian political elections. Palgrave Communications. 2017;5(91). doi:10.1057/s41599-019-0300-3.

3. de Jeude JvL, Caldarelli G, Squartini T. Detecting core-periphery structures by surprise. Europhysics Letters. 2019;125(6):68001.

4. Malvestio I, Cardillo A, Masuda N. Interplay between k-core and community structure in complex networks; 2020.
