# Supplementary material for: Networked partisanship and framing: A socio-semantic network analysis of the Italian debate on migration
Source: PLoS One. 2021 Aug 26;16(8):e0256705. doi: 10.1371/journal.pone.0256705 (PMC8389375; doi:10.1371/journal.pone.0256705)
Supplement: S5 Appendix — (PDF) [file pone.0256705.s005.pdf]

# Networked partisanship and framing: a socio-semantic network analysis of the Italian debate on migration - S5 Appendix

Tommaso Radicioni*[1,2], Fabio Saracco[2], Elena Pavan[3], Tiziano Squartini[2]

**1** Scuola Normale Superiore, P.zza dei Cavalieri 7, 56126 Pisa (Italy)
**2** IMT School for Advanced Studies, P.zza S. Francesco 19, 55100 Lucca (Italy)
**3** University of Trento, via Verdi 26, 38122 Trento (Italy)

*tommaso.radicioni@sns.it

**S5 Appendix. Computing the polarization of non-verified users.** Once the communities of verified users are detected, the procedure to build partisan communities ends with the 'inclusion', within these communities, of non-verified users. In order to do that, the full retweeting network (with all the Twitter users involved in the discussion throughout the entire observation period) is, now, considered. In this network, the information about the community affiliation of the verified users is preserved via a *community label*. A measure of the distribution of the community label of the neighbors is, then, assigned to each non-verified user. This measure is called *polarization index* and determines the balance of the interactions of each non-verified user, in the retweeting network - namely how the portion of interactions of the non-verified users is distributed 'towards' each community of verified users.

Let define $C_c$ (where $c$ represents the community label) as the set of users belonging to community $c$ with whom the non-verified user $\alpha$ has interacted and $N_\alpha$ as the set of neighbors of $\alpha$ with whom he interacted with. The *polarization index* for each non-verified user $\alpha$ is defined as:

$$\rho_\alpha = \max_c \{I_{\alpha c}\} \tag{1}$$

where:

$$I_{\alpha c} = \frac{|C_c \cap N_\alpha|}{|N_\alpha|}. \tag{2}$$

As shown in [1], the polarization index reveals how unbalanced the distribution of retweets, thus providing a clear indication of the target community of each non-verified user. To be sure we include only non-verified users with large values of polarization index, we, first, define a *polarized user* as a non-verified user with a value of the polarization index greater than, or equal to, 0.9. All polarized users are, thus, included within the corresponding community of verified users.

After this first step, we have inferred the orientation of the remaining, non-verified users by propagating the tags of each partisan community obtained for the verified users. A label propagation algorithm, as the one proposed in [2], has been run on the retweeting network: this algorithm implements the idea that each node in the retweeting network joins the same community the majority of its neighbors belongs to. Each verified user is initialized with a unique community label while all the other users (including the polarized ones) have no label; then, these labels propagate through the network until densely connected groups of nodes reach a consensus. In case no majority is found, the algorithm randomly removes a link and re-evaluates the labels [2]. Due to

the stochasticity introduced by this latter step, the label propagation algorithm is repeated 1.000 times. In fact, although this algorithm can modify the label assigned to a non-verified user by the polarization index, labels are still stable after a large number of runs. At the end of such a process, each non-verified user is assigned the community label met with higher frequency across all iterations. After this last step, more than 90% of the Twitter users are assigned to one of the main partisan communities.

The stability of the results of such an algorithm has been already proven in [3] whose authors compare several approaches in order to check the presence of dependencies on the different sets of assumptions. In particular, authors of [3] consider two algorithms, i.e. the Louvain community detection algorithm and the label propagation algorithm proposed in [2], and apply them to three different representations of the retweeting network: the original weighted network of retweets, its unweighted version and the validated projection obtained via the procedure proposed in [4]. The Variation of Information (VI) (i.e., an information theoretic criterion for comparing any two partitions, defined in [5]) is then used to capture the distance among the different partitions. The greatest discrepancies are found between the results of the Louvain algorithm and those of the label propagation algorithm, regardless of the network. In fact, while relatively large VI values are found across the partitions detected by Louvain on the different network representations, the label propagation induces lower VI values across the same partitions.

The aforementioned stability is probably due to several reasons. First, the seeds of the label propagation (i.e., the labels which have fixed values) are themselves extremely stable, as a result of the strict validation procedure. In this sense, the rationale upon which the projection algorithm is based seems to be particularly sound: any two verified users interacting with the same non-verified users are indeed perceived as similar. This interpretation is based on two essential features of the system, i.e. the strong modular structure of the network and the peculiar activity of verified users, much more focused on the production of original messages than on retweeting. Second, due to the adversarial dynamics observed in the present discussion, the label propagation procedure seems not to be sensitive to the topological variation induced by different representations of the retweeting network: indeed, the neighbours of a node are more likely to belong to its community irrespectively of the relative importance of the information carried by the weight.

# References


1. Becatti C, Caldarelli G, Lambiotte R, Saracco F. Extracting significant signal of news consumption from social networks: the case of Twitter in Italian political elections. Palgrave Communications. 2017;5(91). doi:10.1057/s41599-019-0300-3.

2. Raghavan UN, Albert R, Kumara S. Near linear time algorithm to detect community structures in large-scale networks. Physical review E. 2007;76(3):036106.

3. Caldarelli G, de Nicola R, Petrocchi M, Pratelli M, Saracco F. Analysis of online misinformation during the peak of the COVID-19 pandemics in Italy. arXiv e-prints. 2020;.

4. Becatti C, Caldarelli G, Lambiotte R, Saracco F. Extracting significant signal of news consumption from social networks: the case of Twitter in Italian political elections. Palgrave Commun. 2019;doi:10.1057/s41599-019-0300-3.

5. Meilă M. Comparing clusterings by the variation of information. vol. 2777. Springer, Berlin, Heidelberg; 2003. p. 173–187.
