# Supplementary material for: Networked partisanship and framing: A socio-semantic network analysis of the Italian debate on migration
Source: PLoS One. 2021 Aug 26;16(8):e0256705. doi: 10.1371/journal.pone.0256705 (PMC8389375; doi:10.1371/journal.pone.0256705)
Supplement: S6 Appendix — (PDF) [file pone.0256705.s006.pdf]

Networked partisanship and framing: a socio-semantic network analysis of the Italian debate on migration - S6 Appendix

Tommaso Radicioni*[1,2], Fabio Saracco[2], Elena Pavan[3], Tiziano Squartini[2]

1 Scuola Normale Superiore, P.zza dei Cavalieri 7, 56126 Pisa (Italy)
2 IMT School for Advanced Studies, P.zza S. Francesco 19, 55100 Lucca (Italy)
3 University of Trento, via Verdi 26, 38122 Trento (Italy)

*tommaso.radicioni@sns.it

S6 Appendix. Computing the h-index of Twitter users.A useful tool to measure the influence of a given Twitter user, within a discussion, is the well-known Hirsch index (or h-index), widely used to measure the impact of research activity. This index has been also employed for quantifying the ability of Twitter users to produce influential contents via the number of retweets of each of their messages. In [1], h-inde has been used to detect the most influential users during the Brexit debate on Twitter: similarly, we employed it to derive a ranking list of the verified users in the Italian discussion on migration issues. As S3 Table hows, our results further confirm the overall strong personalization of the debate on migration: in fact, Matteo Salvini's Twitter account (*@matteosalvinimi*) has the highest h-index, amounting at more than twice the second ranked value. The presence of several verified accounts belonging to media organizations, such as *@repubblica* and *@Avvenire_Nei*, is another indicator of how the construction of partisan collective identities relates to the activity of these accounts, e.g. in the CSX and the MINGOs communities.

References

1. Grčar M, Cherepnalkoski D, Mozetič I, Kralj Novak P. Stance and influence of Twitter users regarding the Brexit referendum. Computational Social Networks. 2017;4. doi:10.1186/s40649-017-0042-6.
