# Supplementary material for: Networked partisanship and framing: A socio-semantic network analysis of the Italian debate on migration
Source: PLoS One. 2021 Aug 26;16(8):e0256705. doi: 10.1371/journal.pone.0256705 (PMC8389375; doi:10.1371/journal.pone.0256705)
Supplement: S1 Table — (PDF) [file pone.0256705.s007.pdf]

Networked partisanship and framing: a socio-semantic network analysis of the Italian debate on migration - S1 Table

Tommaso Radicioni[*1,2], Fabio Saracco[2], Elena Pavan[3], Tiziano Squartini[2]

1 Scuola Normale Superiore, P.zza dei Cavalieri 7, 56126 Pisa (Italy)
2 IMT School for Advanced Studies, P.zza S. Francesco 19, 55100 Lucca (Italy)
3 University of Trento, via Verdi 26, 38122 Trento (Italy)

*tommaso.radicioni@sns.it

S1 Table. Overview of the most relevant political and mediatic events concerning the Italian Twittersphere discussion about migration.

S1 Table. Overview of the most relevant political and mediatic events for what concerns the Italian Twittersphere discussion about migration.

**Date**	**Event**
May 18th	Italian authorities seize the rescue vessel Sea-Watch 3 after 47 migrants have been rescued.
May 26th	2019 European elections.
Jun 1th	Sea-Watch 3 ends its seizure period.
Jun 7th	The European Council adopts a partial negotiation position about migrants repatriation and new guidelines for security.
Jun 11th	The Italian Council of Ministers approves the ‘Second Security Act’ (‘Decreto Sicurezza Bis’) against illegal immigration and NGOs.
Jun 29th	After two weeks sailing, Sea-Watch 3 enters the Italian territorial waters without any formal permission; its captain Carola Rackete is arrested.
Jul 19th	After her freeing, Carola Rackete goes back to Germany.
Jul 24th	Italian Parliament approves the ‘Second Security Act’ (‘Decreto Sicurezza Bis’).
Aug 1st	The rescue vessel Open Arms rescues 52 migrants.
Aug 9th	The League party submits a no-confidence motion against the Prime Minister Giuseppe Conte. The Italian government crisis starts.
Aug 19th	After the refusal of the Italian government, the Spanish Prime Minister Pedro Sanchez allows Open Arms to disembark migrants in Mallorca.
Sep 2nd	Italian authorities seize the rescue vessel Eleonore.
Sep 5th	The Italian government crisis ends with the Conte-bis cabinet swearing in.
Sep 14th	The rescue vessel Ocean Viking is allowed to disembark 82 rescued migrants in Lampedusa.
Oct 26th	The NGOs involved in search-and-rescue operations at sea meet the new Minister of the Internal Affairs Luciana Lamorgese.
Nov 1st	After seven days sailing, the rescue vessel Alan Kurdi is allowed to dock in Taranto.
