# Supplementary material for: Networked partisanship and framing: A socio-semantic network analysis of the Italian debate on migration
Source: PLoS One. 2021 Aug 26;16(8):e0256705. doi: 10.1371/journal.pone.0256705 (PMC8389375; doi:10.1371/journal.pone.0256705)
Supplement: S2 Table — (PDF) [file pone.0256705.s008.pdf]

Networked partisanship and framing: a socio-semantic network analysis of the Italian debate on migration - S2 Table

Tommaso Radicioni*[1,2], Fabio Saracco[2], Elena Pavan[3], Tiziano Squartini[2]

1 Scuola Normale Superiore, P.zza dei Cavalieri 7, 56126 Pisa (Italy)
2 IMT School for Advanced Studies, P.zza S. Francesco 19, 55100 Lucca (Italy)
3 University of Trento, via Verdi 26, 38122 Trento (Italy)

*tommaso.radicioni@sns.it

S2 Table. Brief description of the main hashtags present in our analysis.

S2 Table. Brief description of the main hashtags present in our analysis.

Hashtag	Translation and context
#salvini	Matteo Salvini is the leader of the right-wing party called the League. He was Minister of the Interior until August 2019 when the League submitted a no-confidence motion against the Prime Minister Giuseppe Conte.
#oceanviking, *#seawatch3*, *#carolarackete*, *#lampedusa*, *#marejonio*	Ocean Viking, Sea Watch 3 and Mare Jonio are names of NGOs (called *ONG* in Italian) rescue boats whose activity is that of providing the first aid to migrants at sea. Lampedusa is an Italian island, approximately at the same distance from Malta, Sicily and the coasts of Tunisia, where rescued migrants have been often disembarked. Carola Rackete is the captain of Sea-Watch 3 who forced the docking ban entering the Italian territorial waters without any formal permission.
#blocconavale	Literally, 'ship block'. Ship blocks represented the strategy used by the Minister of the Internal Affairs Matteo Salvini to forbid NGOs to disembark migrants in Italy.
#portichiusi, *#portiaperti*	Respectively, 'closed ports' and 'open ports'. The first hashtag was used as a support of the Matteo Salvini's political slogan which summarizes his political view about migration policies. The second one has been invented by political adversaries as a reversed version of the initial slogan.
#bibbiano	Bibbiano is a small municipality in Emilia Romagna, a northern Italian region, where a judicial case involved several center-left political actors, as the major of the city, as part of a criminal business for allegedly brainwashing vulnerable children. Although this event is not directly connected with immigration issues, the demand for justice for case victims has been one of the topics of the League and Brothers of Italy against the center-left coalition.
#fateliscendere, *#iostoconcarola*, *#salvinivergogna*, *#salvinihapauradelparlamento* *#salvinidimettiti*, *#ministrodellamalavita*	Respectively, 'let them get off', 'I stand with Carola', 'shame on Salvini', 'Salvini is afraid of the parliament', 'Salvini resign' and 'ministry of the organized crime'. These hashtags were used during the Sea-Watch 3 episode by the CSX, M5S and MINGOs communities to either support Matteo Salvini or Carola Rackete (depending on the position about migration).
#nonfateliscendere, *#iostoconsalvini*, *#salvininonmollare*, *#arrestatecarolarackete*	Respectively, 'don't let them get off', 'I stand with Salvini', 'Salvini don't give up' and 'arrest Carola Rackete'. These hashtags were used by the DX community to show support towards the governmental policies on migration promoted by the Minister of the Internal Affairs at the time, i.e. Matteo Salvini, during the Sea-Watch 3 case.
#clandestini	Literally, 'illegal immigrants'. A term mostly used by Italian right- and far-wing parties to call migrants who land in the Italian coasts.
#facciamorete	Literally, 'let's act as a network'. A political slogan used to call for an antifascist grassroots movement dealing with various civic issues and born to counteract the migration policies of Matteo Salvini.
#governodelfallimento, *#legatifrega*, *#salvinibugiardo*, *#salvinitraditore*	Respectively, 'government of failure', 'League fools you', 'Salvini liar' and 'Salvini traitor'. These hashtags were used by CSX and M5S community to show dissatisfaction towards the behavior of the League and its political leader, leading to the government crisis. The slogan 'government of failure' is a pun linked to the self-proclaimed 'government of change' of the Five Stars Movement and the League.
#crisidigoverno, *governoconte2*	Respectively, 'government crisis' and 'government Conte 2'. These two hashtags were used by M5S community to refer to the political crisis and the newly formed government of the Prime Minister Giuseppe Conte with the support of the Five Stars Movement and the Democratic Party.
#elezionisubito, *#vogliamovotare*	Respectively, 'elections now' and 'we want to vote'. These hashtags were used by the DX community to show their dissatisfaction towards the agreement between the Five Stars Movement and the Democratic Party to form a new government after the crisis.
#dirittiumani, *#inclusione*, *#corridoiumanitari*, *#ioaccolgo*, *#giornatamondialedelrifugiato*	Respectively, 'human rights', 'inclusion', 'humanitarian corridors', 'I host' and 'world refugee day'. These hashtags were used in the CSX and MINGOs communities to discuss about migration issues as search-and-rescue activities, inclusion and human rights at a national and European level.
#decretosicurezzabis	Literally, 'second security act'. This act represents a revision, proposed by the League party, of Italian migration policies introducing fines for NGOs search-and-rescue activities and stricter rules for requesting the residence permits issued for humanitarian reasons.
