# Supplementary material for: Networked partisanship and framing: A socio-semantic network analysis of the Italian debate on migration
Source: PLoS One. 2021 Aug 26;16(8):e0256705. doi: 10.1371/journal.pone.0256705 (PMC8389375; doi:10.1371/journal.pone.0256705)
Supplement: S3 Table — (PDF) [file pone.0256705.s009.pdf]

Networked partisanship and framing: a socio-semantic network analysis of the Italian debate on migration - S3 Table

Tommaso Radicioni*[1,2], Fabio Saracco[2], Elena Pavan[3], Tiziano Squartini[2]

1 Scuola Normale Superiore, P.zza dei Cavalieri 7, 56126 Pisa (Italy)
2 IMT School for Advanced Studies, P.zza S. Francesco 19, 55100 Lucca (Italy)
3 University of Trento, via Verdi 26, 38122 Trento (Italy)

*tommaso.radicioni@sns.it

S3 Table. List of the first ten verified users with the highest values of h-index (computed by considering the activity throughout the entire observation period).

S3 Table. List of the first ten verified users with the highest values of h-index (computed by considering the activity throughout the entire observation period).

Twitter user (screen name)	h-index	Discursive community
matteosalvinimi	231	DX
GiorgiaMeloni	95	DX
LegaSalvini	52	DX
Linkiesta	50	CSX
Capezzone	50	DX
fattoquotidiano	48	M5S
Agenzia_Ansa	43	CSX
Avvenire_Nei	43	MINGOs
repubblica	43	CSX
CarloCalenda	40	CSX
